# Therapy-Induced Neuroplasticity in Chronic Aphasia After Phonological Component Analysis: A Matter of Intensity

**DOI:** 10.3389/fneur.2018.00225

**Published:** 2018-04-09

**Authors:** Karine Marcotte, Laura Laird, Tali Bitan, Jed A. Meltzer, Simon J. Graham, Carol Leonard, Elizabeth Rochon

**Affiliations:** ^1^Centre de recherche de l’Hôpital du Sacré-Coeur de Montréal, Montréal, QC, Canada; ^2^École d’orthophonie et d’audiologie, Faculté de médecine, Université de Montréal, Montréal, QC, Canada; ^3^Department of Speech-Language Pathology, Faculty of Medicine, University of Toronto, Toronto, ON, Canada; ^4^Toronto Rehabilitation Institute – University Health Network, Toronto, ON, Canada; ^5^Department of Psychology, IIPDM, IBBR, University of Haifa, Haifa, Israel; ^6^Rotman Research Institute – Baycrest Centre, Toronto, ON, Canada; ^7^Department of Psychology, University of Toronto, Toronto, ON, Canada; ^8^Heart and Stroke Foundation, Canadian Partnership for Stroke Recovery, Ottawa, ON, Canada; ^9^Department of Medical Biophysics, University of Toronto, Toronto, ON, Canada; ^10^Physical Sciences Platform, Sunnybrook Research Institute, Sunnybrook Health Sciences Centre, Toronto, ON, Canada; ^11^Audiology and Speech-Language Pathology Program, School of Rehabilitation Sciences, University of Ottawa, Ottawa, ON, Canada; ^12^Rehabilitation Sciences Institute, University of Toronto, Toronto, ON, Canada

**Keywords:** aphasia, phonological component analysis, neuroplasticity, functional magnetic resonance imaging, treatment

## Abstract

Despite the growing evidence regarding the importance of intensity and dose in aphasia therapy, few well-controlled studies contrasting the effects of intensive and non-intensive treatment have been conducted to date. Phonological components analysis (PCA) treatment for anomia has been associated with improvements in some patients with chronic aphasia; however, the effect of treatment intensity has not yet been studied with PCA. Thus, the aim of the present study was to identify the effect of intensity on neural processing associated with word retrieval abilities after PCA treatment. We used functional magnetic resonance imaging to examine therapy-induced changes in activation during an overt naming task in two patients who suffered from a stroke in the left middle cerebral artery territory. P1 received intensive PCA treatment whereas P2 received the standard, non-intensive, PCA treatment. Behavioral results indicate that both standard and intensive conditions yielded improved naming performance with treated nouns, but the changes were only significant for the patient who received the intensive treatment. The improvements were found to be long lasting as both patients maintained improved naming at 2-months follow-ups. The associated neuroimaging data indicate that the two treatment conditions were associated with different neural activation changes. The patient who received the standard PCA showed significant increase in activation with treatment in the right anterior cingulate, as well as extensive areas in bilateral posterior and lateral cortices. By contrast, the patient who received intensive PCA showed more decreases in activation following the treatment. Unexpectedly, this patient showed subcortical increase in activation, specifically in the right caudate nucleus. We speculate that the recruitment of the caudate nucleus and the anterior cingulate in these patients reflects the need to suppress errors to improve naming. Thus, both short-term intensive and standard, non-intensive, PCA treatment can improve word retrieval in chronic aphasia, but neuroimaging data suggest that improved naming is associated with different neural activation patterns in the two treatment conditions.

## Highlights

○ We report on two participants who benefited from PCA treatment.
○ We examined therapy-induced processing using an overt naming task during fMRI.
○ We tested the effects of intensive and standard (non-intensive) PCA treatment.
○ Results indicate that both standard and intensive conditions yielded improved naming performance.
○ Naming improvements were associated with different neural changes in the two treatment conditions.

## Introduction

According to the most recent Cochrane review (1), high-intensity aphasia therapy leads to reduced aphasia severity and greater functional improvement in communication than low-intensity therapies, but may lead to a higher dropout rate. Recently, Breitenstein et al. (2) reported a multicenter randomized controlled trial in which they showed that 3 weeks of intensive (i.e., ≥10 h per week) speech and language therapy significantly improved verbal effectiveness in everyday life situations. Interestingly, none of their 158 participants dropped out of the study, which mitigates the concern raised by the latest Cochrane review (1). Despite growing evidence regarding the importance of intensity and dose in aphasia therapy, patients with aphasia receive far less than what is considered intensive therapy in clinical settings (3).

Several studies have compared intensive and less intensive aphasia treatment behaviorally [e.g., Ref. (4–6)], but no study has investigated the neural basis of intensity associated with a specific treatment. Since intensity may be an important factor in maximizing neuroplasticity in recovery from brain damage, a study contrasting the effects of intensive and non-intensive treatment is required. Preliminary to such an undertaking in substantial patient cohorts, we used functional magnetic resonance imaging (fMRI) to investigate the neural changes associated with anomia treatment in two patients: one who received short-term intensive treatment and another who received non-intensive (i.e., “standard”) treatment. Importantly, both patients received the same amount of therapy overall and both received the same treatment for anomia, the phonological components analysis (PCA) treatment approach.

Phonological components analysis is a sound-based therapy in which participants are asked to identify five phonological components (e.g., rhymes with?; first sound?) of a word they cannot name, guided by the use of a chart. We and others have shown that PCA treatment improves naming performance in patients who suffer from chronic non-fluent or fluent aphasia (7–9) following a left hemisphere stroke. Improved naming after standard PCA treatment has been associated with neural activation changes in left hemisphere areas (e.g., supramarginal gyrus, left inferior frontal gyrus) and to a lesser extent, in right hemisphere areas (e.g., precuneus) (10, 11).

Thus, the aim of this case report is to use this well-established specific treatment to better understand the brain plasticity mechanisms associated with the intensity of delivery of aphasia treatment.

## Case History

P1 is a 59-year-old right-handed woman who developed Broca’s aphasia following a stroke involving the left middle cerebral artery (MCA) territory 3 years prior to the present study. She worked as a teacher and in administration. P2 is a 58-year-old right-handed male who developed Broca’s aphasia following a stroke in the left MCA territory 1 year before the study. He had hypertension, coronary artery disease, and dyslipidemia. The patient worked in television production prior to the stroke. Both patients were monolingual English speakers, wear reading glasses, and passed a hearing screening test. There was no history of memory loss or any other neurological disorder at the time of stroke.

**Figure 1:**
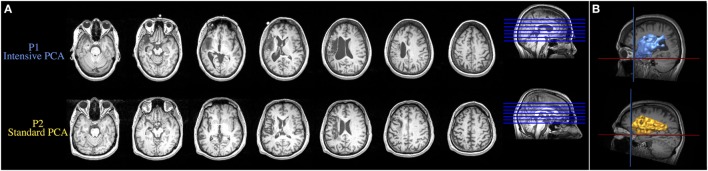
**(A)** Axial MRI images showing the lesions resulting from a left ischemic stroke in the middle cerebral artery territory. **(B)** Reconstruction in 3D of the brain lesion of both patients. Upper and lower images are from the patient who received the intensive condition and the patient who received the standard regimen, respectively.

Structural MRI revealed a stroke in the left MCA territory for both patients. Axial MRI images in Figure 1A show the lesions of both patients and reconstructions in 3D of the lesions are presented in Figure 1B. The neurological examination of both patients also revealed right hemiparesis and right hypoesthesia. Both patients participated in a larger group study investigating the behavioral differences in treatment outcomes associated with intensive versus standard PCA treatment (12). Both patients provided free and informed consent to participate in the experiments, which were conducted with the approval of the Research Ethics Board at Baycrest Centre, Toronto, ON, Canada. Written informed consent was obtained from both participants for the publication of these case reports.

## Materials and Methods

### Overall Experimental Procedure

Patients were randomly assigned to treatment condition. P1 was assigned to the intensive treatment, which consisted of 10 sessions of 3 h each, given over the course of 2.5 weeks, whereas P2 was assigned to the standard treatment, which consisted of 30 sessions of 1 h each, given over the course of 10 weeks. A baseline language assessment was conducted prior to treatment, followed by an initial fMRI session (“pre” timepoint), which served to identify the neural substrate of spontaneous correct naming. Afterward, patients received PCA treatment (7). A second fMRI session (“post” timepoint) was performed at the end of 30 h of PCA for both patients. This occurred after 2.5 weeks (10 sessions) for the intensive regimen and after 10 weeks (30 sessions) for the standard regimen. During both fMRI sessions, patients performed an overt naming task.

### Baseline Language Assessment

We used a series of tests to assess the different aspects of language abilities before PCA treatment began. The assessment included the following tests: the Boston Naming Test (13), the minimal feature match sub-test of the Birmingham Object Recognition Battery (14), and the Western Aphasia Battery (15). Table 1 shows the performance of both patients on this battery.

**Table 1:** Inclusion screening measures before phonological components analysis treatment.

	P1 (intensive)	P2 (standard)
Boston Naming Test (/60)	16 (27%)	14 (23%)
Birmingham Object Recognition Battery (/25)	24 (96%)	25 (100%)
Western Aphasia Battery		
Language quotient	68%	70%
Aphasia quotient	66%	62%

### PCA Therapy and fMRI Task Design

Stimuli for the naming task were color pictures presented on a white background. To provide individualized treatment, stimuli for PCA treatment were selected on the basis of individual performance on the baseline evaluation. Therefore, each patient was treated with different stimuli. Two sets of words were created for each patient: of the 60 incorrectly named words, only 30 were treated. Words were pseudo randomly assigned to either the treated or the untreated condition.

The PCA treatment was conducted in multiple sessions according to the procedures described in Leonard et al. (7). Briefly, the examiner presents a target picture in the center of a chart and asks the patient to name the picture. Then, the patient provides five phonological components (a rhyming word/the first sound/another word that starts with the same first sound/the last sound/the number of syllables) of the target picture. Once completed, the patient tries to name the target once again. Then the examiner reviews all five phonological components and the patient tries to name the target a third time.

Treatment under the short-term intensive condition was provided for patient P1 over a period of 2.5 weeks. Four treatment sessions occurred in the first 2 weeks, followed by two sessions in the last week, for approximately 3 h per session. Ten-minute breaks were incorporated every hour. Patient P2 received the standard treatment intensity over a period of 10 weeks, in three sessions per week that each lasted approximately 1 h. Thus, both patients received a total of 30 h of therapy.

The fMRI sessions used an event-related design. The 192 experimental stimuli (from which 48 were treated, including 18 words that were repeated) were presented in three separate runs. Stimuli were presented for 4,500 ms, with an interstimulus interval ranging from 3,130 to 13,615 ms. Participants were instructed to name each picture, as clearly and accurately as possible, while avoiding head movements. An MRI-compatible microphone was placed close to the participant’s mouth, and Audacity^1^ was used to record oral responses.

Visual stimuli were delivered using standard software (Presentation software v.15.0^2^) from a computer onto a projection screen at the front of the magnet bore. Patients viewed the projection screen through an angled mirror attached to the head coil.

### Imaging Protocol

Anatomical and functional MR images were acquired using a 3.0 T MRI system (Magnetom Trio, Siemens, Erlangen, Germany) with a standard 8-channel head coil. Patients lay supine on the MRI patient table with their head stabilized by foam padding. First, a high-resolution structural image was obtained using a 3D T1-weighted magnetization prepared rapid gradient echo imaging [repetition time (TR) = 2,000 ms; echo time (TE) = 2.63 ms; 160 slices; matrix = 256 × 256; voxel size = 1 mm isotropic; field of view (FOV) = 256 mm]. Functional MRI was performed using T2*-weighted echo planar imaging (TR = 2,300 ms; TE = 30 ms; 38 slices; matrix = 64 × 64; FOV = 200 mm; flip angle = 90°; slice thickness = 3.5 mm).

### fMRI Analysis

Preprocessing and statistics were performed using SPM12 freeware (Wellcome Department of Imaging Neuroscience, London, UK) (16). Each run was preceded by 20 s of rest, which were discarded to eliminate non-steady state magnetization artifacts. Preprocessing included slice timing, realignment, segmentation, normalization, and spatial smoothing using a 6 mm full-width-at-half-maximum Gaussian filter. Analyses were performed to separate blood-oxygen-level dependent (BOLD) responses for each trial type. For each participant, task-related BOLD changes were examined by convolving a vector of naming onset with the hemodynamic response function and its temporal derivative.

Preprocessed data were analyzed using the general linear model implemented in SPM12. Statistical parametric maps were obtained for each individual participant, by applying linear contrasts to the parameter estimates for the events of interest; this resulted in a *t*-statistic for every voxel. Individual maps were calculated for each condition of interest by employing a one-sample *t*-test without constant term (random effects) on the resulting contrast image. Two main contrasts [(naming treated words before PCA > naming treated words after PCA) and (naming treated words after PCA > naming treated words before PCA)] were performed with a cluster size (k) threshold >5 voxels and *p* < 0.001 (uncorrected). Optimal anatomical localization was based on the Montreal Neurological Institute (MNI) template brain of SPM12 as well as on Talairach coordinates (17) which were obtained using a non-linear transformation^3^ (18) and labeled using the Talairach Daemon applet^4^ (19, 20). The coordinates are reported in Table 2 are SPM12 (MNI) coordinates.

**Table 2:** Main peak of activation for the [naming treated words before phonological components analysis (PCA) > naming treated words after PCA] contrast and the (naming treated words after PCA > naming treated words before PCA) contrast.

Left							Right						
Region	BA	*x*	*y*	*z*	*T*-score	Cluster size	Region	BA	*x*	*y*	*z*	*T*-score	Cluster size
**Decrease in activation during treatment (naming treated words before PCA > naming treated words after PCA)**													

**P1 (intensive PCA)**													
Precentral gyrus	4	−30	−24	60	3.87	12	Posterior cingulate	30	16	−66	8	3.67	31
Medial frontal gyrus	6	−4	−20	60	3.37	8	Precuneus	7	10	−52	54	3.48	10
**P2 (standard PCA)**													
Middle temporal gyrus	21	−62	−42	−10	3.63	6	Postcentral gyrus	43	64	−4	12	3.82	33
Precentral gyrus	6	−36	0	36	3.57	6							
Putamen		−24	18	2	3.43	5							

**Increase in activation during treatment (naming treated words after PCA > naming treated words before PCA)**													

**P1 (intensive PCA)**													
Medial frontal gyrus	11	−8	38	−16	3.24	7	Caudate		4	14	−2	3.27	9
**P2 (standard PCA)**													
Superior parietal lobule	7	−28	−54	64	5.50	130	Superior parietal lobule	19/7	20	−86	36	4.92	1,818
Inferior frontal gyrus	47	−30	34	−18	4.76	79	Anterior cingulate	25	2	8	−6	4.49	386
Cuneus	19	−12	−86	38	4.12	67	Inferior frontal gyrus	47	28	24	−18	4.35	91
Middle frontal gyrus	10/11	−26	56	12	3.96	37	Postcentral gyrus	40	40	−36	64	4.32	38
Inferior frontal gyrus	47	−44	30	−16	3.86	11	Precentral gyrus	4	32	−28	52	4.25	41
Cuneus	18/19	−14	−96	26	3.81	67	Fusiform gyrus	37	58	−54	−2	4.22	129
Middle occipital gyrus	18	−34	−90	6	3.64	12	Precentral gyrus	6	28	−16	60	4.09	56
Inferior parietal lobule	40	−48	−46	56	3.62	6	Postcentral gyrus	40	56	−28	38	4.05	56
Middle occipital gyrus	18	−28	−82	−4	3.56	5	Paracentral lobule	31	8	−34	46	4.04	89
Superior temporal gyrus	39/22	−56	−60	14	3.54	17	Cerebellum (declive)		50	−50	−30	3.88	17
Superior temporal gyrus	39	−44	−48	4	3.53	8	Orbital gyrus	11	4	46	−20	3.71	19
Superior temporal gyrus	39	−48	−58	12	3.49	5	Cerebellum (tonsil)		22	−30	−44	3.65	16
Cerebellum (culmen)		−38	−50	−38	3.48	5	Inferior frontal gyrus	47	50	32	−10	3.64	8
Medial frontal gyrus	10/11	−12	44	−12	3.44	6	Middle temporal gyrus	19	38	−82	18	3.53	11
Middle temporal gyrus	37	−48	−66	−2	3.42	24	Inferior parietal lobule	39	50	−62	42	3.52	46
Middle occipital gyrus	19	−38	−88	20	3.35	7	Superior frontal gyrus	10	22	62	0	3.42	6
Thalamus		−10	−34	6	3.34	8	Precuneus	31	10	−52	34	3.38	8
Superior frontal gyrus	10/11	−14	56	−12	3.30	5	Paracentral lobule	5	2	−36	54	3.38	9
							Sub-gyral	7	26	−50	58	3.38	5
							Inferior parietal lobule	40	54	−50	48	3.37	16
							Precentral gyrus	4/10	44	−20	50	3.33	5
							Anterior cingulate	32	6	30	−10	3.32	7
							Inferior parietal lobule	40	54	−46	30	3.32	9
							Superior temporal gyrus	41	52	−30	14	3.24	7

*Coordinates represent voxels significant at *p* < 0.001, uncorrected for multiple comparisons across the whole brain (*t* > 3.14)*.

## Results

### Language Assessments

Improvement following treatment was observed for treated words, but not for untreated words (see Figure S1 in Supplementary Material). Although both patients improved in their production of treated words, the change was only significant for P1 (McNemar Change Test, *p* = 0.007), treated in the intense condition and not for P2 (McNemar Change Test *p* = 0.210), who was treated in the standard condition. Interestingly, for both patients, posttreatment performance was maintained 4 weeks as well as 8 weeks after therapy (Figure S1 in Supplementary Material).

### Changes in fMRI Activity Patterns

**Figure 2:**
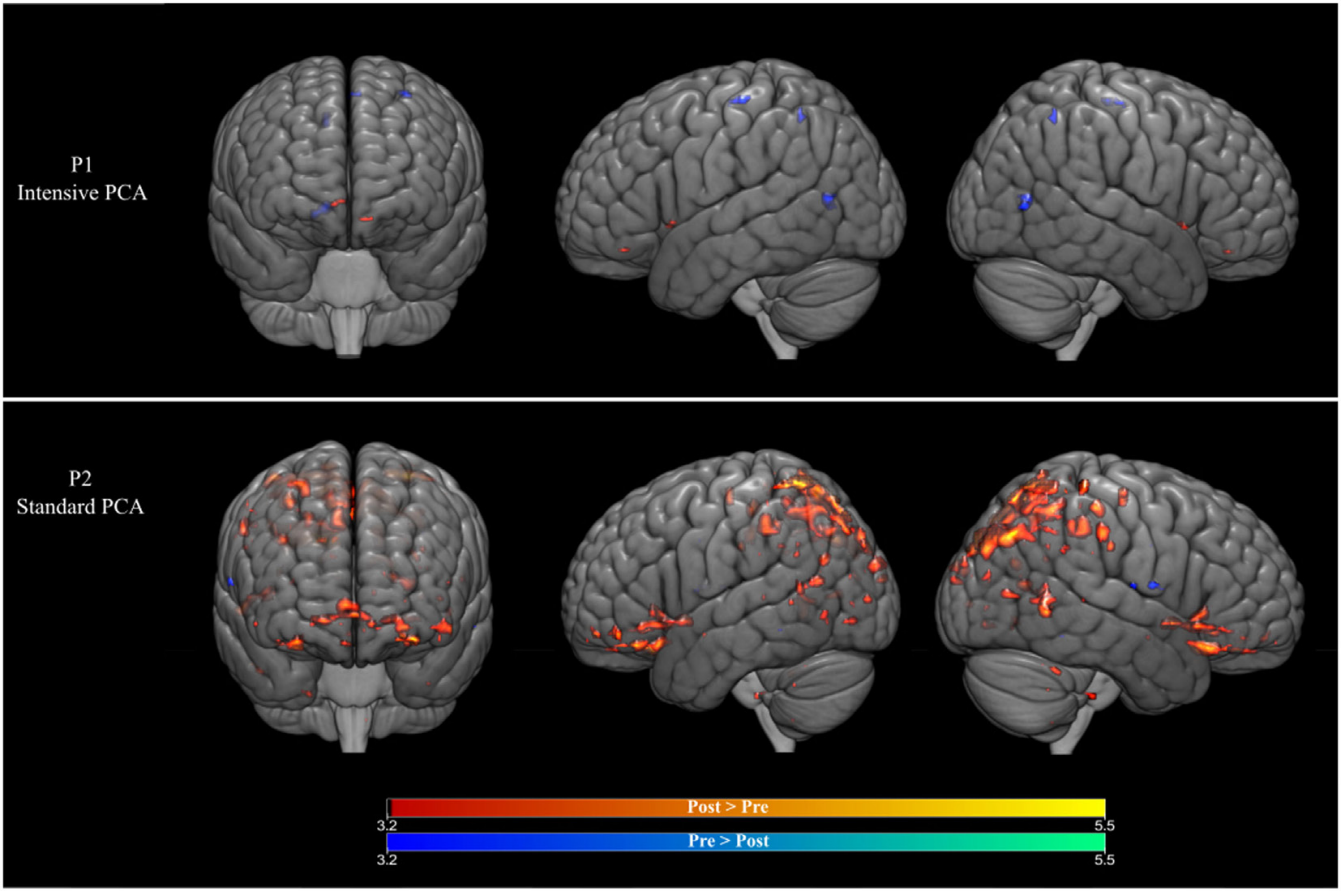
(Top row) Brain activation changes for participant P1 who received the intensive phonological components analysis (PCA). (Bottom row) Brain activation changes for participant P2 who received the intensive PCA. Decreases in activation during treatment are shown in blue, whereas increases in activation are shown in red (*p* < 0.001, >5 contiguous voxels).

For P1 (who received intense PCA treatment), activation during naming of treated words increased during PCA treatment (post > pre) in the right caudate nucleus, as well as in the left medial frontal gyrus. A decrease in activation during treatment (pre > post) was found in the right posterior cingulate gyrus, as well as the precentral gyrus and the medial frontal gyrus in the left hemisphere. Changes were also observed in the precuneus in the right hemisphere (see Figure 2 and Table 2).

For P2 (who received standard PCA treatment), activation during naming of treated words increased during PCA treatment (post > pre) in the superior temporal gyrus bilaterally, as well as the right precentral gyrus and the left inferior frontal gyrus. A decrease in activation during treatment (pre > post) was found in the middle temporal gyrus, the precentral and the putamen in the left hemisphere, and the postcentral gyrus in the right hemisphere.

## Discussion

Similar to results obtained in our previous studies (7, 10), language production by two patients with chronic non-fluent aphasia was improved by PCA, although the change was only significant for the patient in the intensive condition. The improvements were also found to be long lasting as both patients showed maintained treatment effects at 1- and 2-month follow-ups. The associated fMRI data indicate that improved naming performance for treated words was mainly associated with decreases in activation following therapy for the patient who received intensive treatment, whereas the patient who received the standard PCA treatment showed increased cortical activity bilaterally.

Decrease of activation has been associated with more efficient processing following therapy in patients suffering from aphasia (21–23). Consistent with this evidence, the present results suggest that the active engagement of the participant who received the intensive PCA treatment was associated with more decreases than increases in activation, which we attribute to more efficient processing. It should be noted that only P1 who received the intensive treatment showed a significant improvement in naming during treatment, and this improvement was obtained in a very short period of time (2.5 versus 10 weeks, respectively). These results confirm previous findings that treatment related changes in activation can be observed within a very short period of time (24–26).

By contrast, increased neural activity in distributed bilateral areas was observed for participant P2 who received the standard regimen. Greater increases of activation may be due to less efficient or malfunctioning processing (21). Participant P2 who received the standard PCA treatment may have improved his naming abilities, by having more/stronger increases, but his modest improvement could be due to persistently less efficient processing in less specific language areas (21–23).

We note that increase of activation was also found in participant P1. It was somewhat unexpected initially to find that the activation changes for the patient who received intensive PCA were observed in the right caudate nucleus. However, a growing body of evidence suggests that the caudate nucleus is involved in cognitive control, including language control processing (27, 28) and in particular in the selection of the most relevant language features and the inhibition of irrelevant ones. In a recent study, Gronholm et al. (29) reported that 15% of their patient participants presenting with language impairments had focal lesions of the caudate nucleus and adjacent white matter. This finding supports the growing body of evidence which indicates that the caudate nucleus is involved in procedural learning in language tasks (30), which may be associated with automaticity. Similarly, a significant increase in activation was observed for participant P2 in the right anterior cingulate, which is also involved in the suppression of irrelevant words in language production (31). We speculate that the recruitment of the caudate nucleus and the anterior cingulate reflects the need to suppress errors to improve naming ability.

The present work has some limitations. The results from these cases serve as a starting point for investigating the effects of treatment intensity. These findings need to be replicated with larger sample sizes (32), while also bearing in mind that one potential limitation of group analyses in stroke patients is the variability that characterizes both the language impairments and the location of the stroke (33). Accordingly, previous group studies using fMRI have reported substantial variability for individual patients with aphasia [e.g., Ref. (11, 26, 34)]. Another potential caveat of these case studies is the sex difference between the two participants. Although some studies have found that language lateralization seems to be similar in males and females (35–39) with many differences more driven by age (39), it must be acknowledged that a lack of consensus exists regarding the neural correlates of sex/gender differences in language processing (40) and future studies should consider this factor. Regarding treatment outcome, the improvement was not statistically significant for P2. It is possible that changes in activation for this patient reflected random inter-session variance. However, P2 maintained his posttreatment performance, including the difference between his treated and untreated words, at both follow-up sessions, which argues against this possibility. In addition, the present analyses were based on both correctly and incorrectly named words, as the number of words correctly named was not sufficient for distinct separate analysis. Finally, the functional changes observed in the present study may be related to the effect of practice on the task during scanning (41), rather than the therapy. However, overt naming tasks using overtrained pictures are generally considered to involve a decrease of activation and can still be suitable for longitudinal studies in poststroke aphasia as long as overtrained pictures are randomly mixed with novel (e.g., untreated) stimuli (42), as was the case in our study.

## Concluding Remarks

In conclusion, both short-term intensive and standard, non-intensive, PCA treatment improved word retrieval of two chronic aphasia patients. The associated neuroimaging data suggest that improved naming is associated with different neural activation patterns in the two treatment conditions.

## Ethics Statement

Both patients provided free and informed consent to participate in the experiments, which were conducted with the approval of the Research Ethics Board at Baycrest Centre, Toronto, ON, Canada. Written informed consent was obtained from both participants for the publication of these case reports.

## Author Contributions

KM, LL, JAM, CL, and ER conceived and designed the experiments. KM, LL, TB, JAM, SJG, CL and ER analyzed, interpreted the data and wrote the paper. CL and ER supervised the study.

## Conflict of Interest Statement

The authors declare that the research was conducted in the absence of any commercial or financial relationships that could be construed as a potential conflict of interest.

## Footnotes

^1^http://audacity.sourceforge.net/?lang=fr (Accessed: November, 2017).

^2^www.neurobs.com (Accessed: January, 2015).

^3^www.bioimagesuite.org/Mni2Tal/index.html (Accessed: February, 2018).

^4^http://www.talairach.org/applet.html (Accessed: February, 2018).

## Supplementary Material

Figure S1 Accuracy of production of treated and untreated words as a function of treatment condition.
