# Supplementary figures and images for: Therapy-Induced Neuroplasticity in Chronic Aphasia After Phonological Component Analysis: A Matter of Intensity
Source: Front Neurol. 2018 Apr 9;9:225. doi: 10.3389/fneur.2018.00225 (PMC5900891; doi:10.3389/fneur.2018.00225)

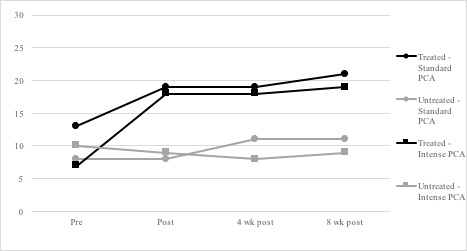

Supplement: Figure S1 — Accuracy of production of treated and untreated words as a function of treatment condition. [file image_1.jpeg]
